# Population genomics of pearl millet (*Pennisetum glaucum* (L.) R. Br.): Comparative analysis of global accessions and Senegalese landraces

**DOI:** 10.1186/s12864-015-2255-0

**Published:** 2015-12-09

**Authors:** Zhenbin Hu, Bassirou Mbacké, Ramasamy Perumal, Mame Codou Guèye, Ousmane Sy, Sophie Bouchet, P. V. Vara Prasad, Geoffrey P. Morris

**Affiliations:** Department of Agronomy, Kansas State University, Manhattan, KS 66506 USA; Ecole Nationale Supérieure d’Agriculture, Université de Thiès, Thiès, BP 296 Senegal; Agricultural Research Center—Hays, Kansas State University, Hays, KS 67601 USA; Institut Sénégalais de Recherches Agricoles, Thiès, BP 3320 Senegal

**Keywords:** Crop diversity, Genotype-by-sequencing, Population structure, Principal component analysis, Single nucleotide polymorphism

## Abstract

**Background:**

Pearl millet is a staple food for people in arid and semi-arid regions of Africa and South Asia due to its high drought tolerance and nutritional qualities. A better understanding of the genomic diversity and population structure of pearl millet germplasm is needed to support germplasm conservation and genetic improvement of this crop. Here we characterized two pearl millet diversity panels, (i) a set of global accessions from Africa, Asia, and the America, and (ii) a collection of landraces from multiple agro-ecological zones in Senegal.

**Results:**

We identified 83,875 single nucleotide polymorphisms (SNPs) in 500 pearl millet accessions, comprised of 252 global accessions and 248 Senegalese landraces, using genotyping by sequencing (GBS) of *PstI*-*MspI* reduced representation libraries. We used these SNPs to characterize genomic diversity and population structure among the accessions. The Senegalese landraces had the highest levels of genetic diversity (π), while accessions from southern Africa and Asia showed lower diversity levels. Principal component analyses and ancestry estimation indicated clear population structure between the Senegalese landraces and the global accessions, and among countries in the global accessions. In contrast, little population structure was observed across in the Senegalese landraces collections. We ordered SNPs on the pearl millet genetic map and observed much faster linkage disequilibrium (LD) decay in Senegalese landraces compared to global accessions. A comparison of pearl millet GBS linkage map with the foxtail millet (*Setaria italica*) and sorghum (*Sorghum bicolor*) genomes indicated extensive regions of synteny, as well as some large-scale rearrangements in the pearl millet lineage.

**Conclusions:**

We identified 83,875 SNPs as a genomic resource for pearl millet improvement. The high genetic diversity in Senegal relative to other regions of Africa and Asia supports a West African origin of this crop, followed by wide diffusion. The rapid LD decay and lack of confounding population structure along agro-ecological zones in Senegalese pearl millet will facilitate future association mapping studies. Comparative population genomics will provide insights into panicoid crop evolution and support improvement of these climate-resilient crops.

**Electronic supplementary material:**

The online version of this article (doi:10.1186/s12864-015-2255-0) contains supplementary material, which is available to authorized users.

## Background

Ensuring food security in the world’s arid and semi-arid regions is a great challenge due to rapid population growth and strong effects of climate change in these regions [1]. Food security in these regions would be strengthened by higher crop yields, greater yield stability, and increased macro- and micro-nutrient quality of staple crops [2]. Pearl millet is an important cereal crop for arid and semi-arid regions of Africa and south Asia due to its high nutritional value and its exceptional tolerance to drought and high temperature. Pearl millet is also important forage and stover crop [3]. Pearl millet has higher nutritional quality than many other cereal grains in terms of minerals and macronutrient quality [4]. As an orphan crop, the potential of pearl millet for climate-resilient agriculture has yet to be fully realized [5, 6].

**Table 1:** SNPs identified by genotyping by sequencing in 500 pearl millet accessions

SNPs	No. of every SNP type	Caused by	Total No. of transition and transversion
A/G	28769	Transition	53208
C/T	24439		
A/C	6832	Transversion	30667
G/T	12016		
A/T	4315		
G/C	7504		

Pearl millet belongs to the Panicoideae subfamily of grasses (*Poaceae* family) and was domesticated over 4500 years ago from *Pennisetum glaucum* ssp. *monodii* [7–9], Most research puts the origins of pearl millet somewhere along Sahelian zone from Senegal to Sudan [7–12], likely around present-day Mali [9]. Pearl millet later diffused into eastern Africa, southern Africa and South Asia, and is now cultivated about 28 million hectares worldwide with grain production of more than 22 million tons a year [3]. In Senegal, the westernmost country of the African mainland, pearl millet is the most important staple cereal, with about 1 million hectares cultivated and about 0.5 million tons of grain produced each year [13]. Despite the value of pearl millet to food security in Senegal, there is relatively little known about the genetic diversity of pearl millet in Senegal compared to other Sahelian countries [14, 15].

Understanding genomic diversity and population structure of crop germplasm is important for germplasm conservation, cultivar development, and quantitative trait loci (QTL) mapping, and lays the foundation for genomics-assisted breeding [16, 17]. Historically, genetic characterization of pearl millet has lagged behind other cereals [3, 6]. To address this gap, several linkage maps have been constructed, and QTL mapping studies have been carried out on grain yield, stover yield, height, biotic stress, and abiotic stress traits [18–25]. High density markers are also essential for fine mapping QTL, for use in molecular breeding or marker-assisted selection [26]. Several studies have been conducted to understand the genetic diversity of pearl millet germplasm using RFLP markers [27], AFLP markers [14, 28], and SSR markers [15, 29–32]. These studies illuminated key features of population structure and diversity, but were targeted to germplasm from a specific regions [15, 29], or were conducted on global germplasm but with low marker density [11, 27]. Thus, genome–wide characterization of worldwide pearl millet germplasm is needed to gain a more complete understanding of genomic resource in this crop.

Next generation sequencing technologies have been used in many crops to rapidly identify genomic variation and develop genetic maps [33–36]. In order to take advantage of sequencing to genotype large populations, reduced representation sequencing methods have been developed, such as restriction site-associated DNA sequencing (RAD-seq) and genotyping-by-sequencing (GBS) [37–39]. These methods involve digestion of genomic DNA with specific restriction enzymes, ligation of unique barcoded adapters, and sequencing of pooled libraries [37–39]. GBS can provide high density SNP discovery and genotyping for large sets of diverse individuals at very low cost, with or without a reference genome sequence [38]. GBS has been used to genotype diverse germplasm and facilitate genome-wide association studies in many crops including maize, wheat, barley, sorghum, switchgrass, and soybean [40–45]. Recently, a high-density genetic map in pearl millet was constructed using GBS on a biparental population [46], but a GBS survey of diverse germplasm in pearl millet has not yet been described.

Here, we report the application of GBS to 500 pearl millet accessions, which include Senegalese landraces and diverse global accessions. A total of 83,875 SNPs were discovered and used to characterize genomic diversity and population structure in this species. These resources lay the foundation for future genome-wide association studies and genomics-assisted improvement of pearl millet.

## Results

### Genome-wide SNP data from global germplasm

**Fig. 1:**
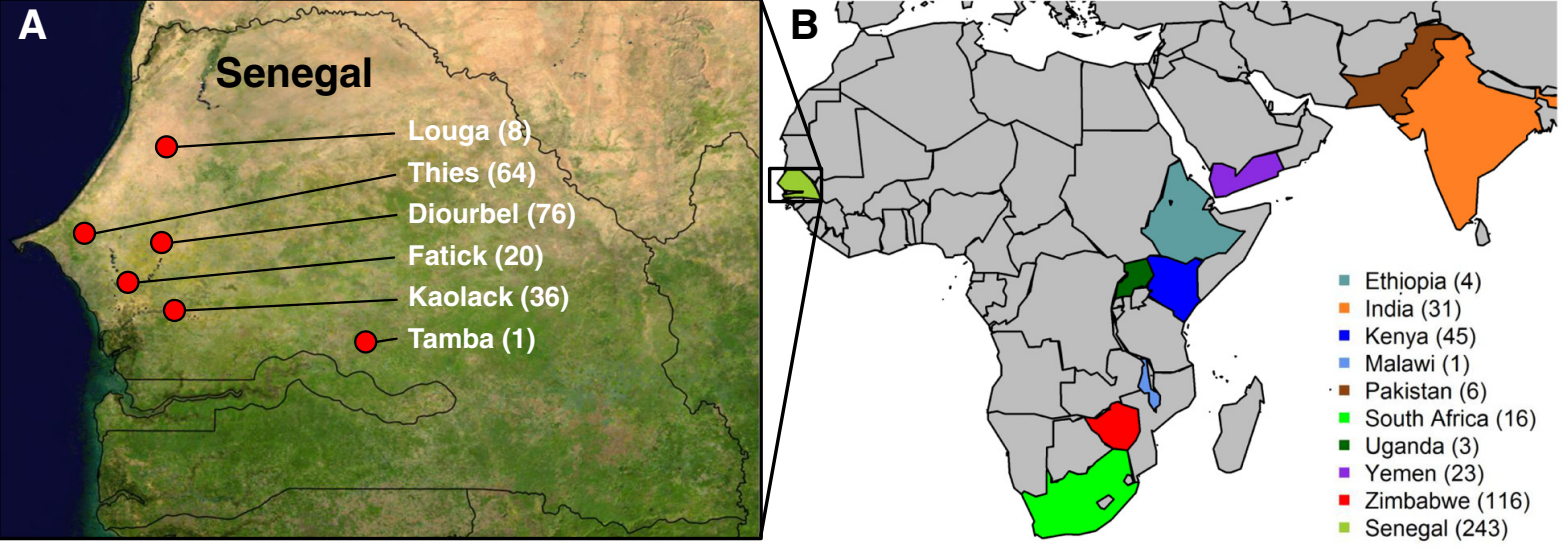
The distribution of pearl millet accessions used in the study. **a** The distribution of 243 Senegalese pearl millet accessions. The red points represent the regions in Senegal where pearl millet landraces were collected, with region name and number of landraces noted. An additional 38 Senegalese pearl millet landraces were without origin information in Senegal. **b** The country-level origins of the global pearl millet accessions

Two pearl millet germplasm sets were genotyped with GBS in present study. One set was 249 pearl millet accessions (named “Senegalese landraces” hereafter) contributed by Senegalese Institute of Agricultural Research (Institut Sénégalais de Recherches Agricoles; ISRA). These accessions originated from farmer cooperators in agro-ecological regions across Senegal, including West-Central Agricultural Region (Louga, Thies, and Diourbel), Agricultural Expansion Region (Tamba), Saloum Agricultural Region (Kaolack), and the Estuary Region (Fatick and Kaolack) (Fig. 1a) [47] of Senegal (Fig. 1a; Additional file 1: Table S1). All accessions were landraces, excepted 11 improved or introduced accessions, including six introduced lines from Mali, Niger, and Mauritania (Additional file 1: Table S1). While these Agricultural regions of Senegal are all part of the major pearl millet growing zone in Senegal, the annual precipitation ranges from 400 mm year^−1^ (Louga) to 770 mm year^−1^ (Tamba). Another set was 262 global pearl millet accessions selected from United States Department of Agriculture (USDA) National Plant Germplasm System (NPGS) (http://www.ars-grin.gov) based on global distribution (“global accessions” hereafter) (Fig. 1b). The global accessions originate from Africa, Asia, and the Americas, with the greatest sampling from Zimbabwe, Kenya, India, Yemen, and South Africa (Fig. 1b, Additional file 1: Table S1).

**Fig. 2:**
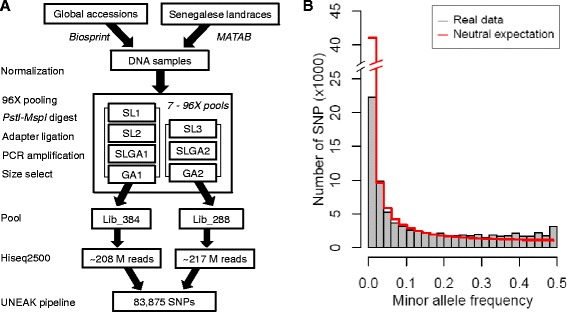
Pearl millet SNP discovery and allele frequency. **a** Flowchart of genotyping-by-sequencing for 511 pearl millet accessions. “SL” represents the pools with Senegalese landraces, “GA” represents the pools with global accessions, and “SLGA” represents the pools with accessions from both germplasm sets. SL3 had the same accessions as SL2 but the library preparation failed. For SLGA2, Senegalese landraces were duplicates of the Senegalese landraces in SLGA1, but the sequence quality was low. **b** The minor allele frequency distributions of observed SNPs (*gray bar*) compared to the neutral allele frequency spectrum (*red line*) in the 500 pearl millet accessions

Two lanes of single-end Illumina HiSeq 2500 were used to sequence two libraries, one 384X multiplexed library (“Lib_384”) and one 288X multiplexed library (“Lib_288”), and generate a total of ~110 GB of raw data (Fig. 2a). Two libraries (two lanes) generated approximately 4.25 × 10^8^ reads (208,404,899 for Lib_384 and 216,646,749 for Lib_288), corresponding to an average of 0.64 million reads per individual. For Lib_384, there were 184,113,400 reads with the barcode and cut site overhang (88.3 %), and for Lib_288 there were 84,493,320 reads (39.0 %) with a good barcode and cut site overhang, for an overall average of 63.6 % reads with a good barcode. After merging, 18,126,075 tags were generated, and 1,460,464 tags were retained after filtering, which were covered by 246,284,116 matching reads. Totally, 83,875 SNPs were identified across all 511 pearl millet accessions with average depth of 1264 tags per site, across all samples (Additional file 2: Table S2). To find the reason for the low percentage of good barcode and overhang in Lib_288, we called SNP for two libraries without merging the duplicated samples in Lib_384 and Lib_288. We found two libraries which were pooled into the Lib_288 which had either failed (SL3) or had low quality (SLGC2) (Fig. 2a). Finally, 11 pearl millet accessions were dropped because of poor data quality, leaving 500 pearl millet accessions (252 global accessions and 248 Senegalese landraces) for further analysis.

Analysis of the SNPs showed 63.4 % (53,208/83,875) of the nucleotide changes were transitions, while 36.5 % (30,667/83,875) of the SNPs were transversions (Table 1). The observed transition:transversion ratio is 1.74. The minor allele frequency (MAF) distribution was close to the neutral expectation, except for a paucity of rare alleles (MAF < 0.02) and a slight excess of alleles with ~50 % frequency (MAF > 0.48) (Fig. 2b). The minor allele frequency distribution of Senegalese landrace was closer to the neutral allele distribution than that of global accessions (Additional file 3: Figure S2A and B).

### Genetic diversity and relatedness

**Fig. 3:**
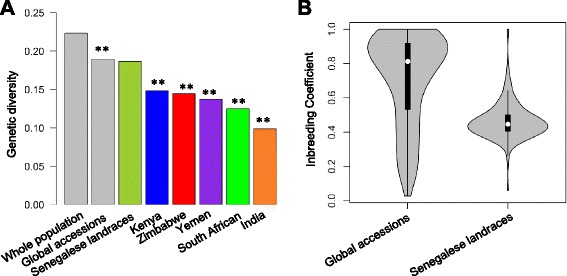
Genome-wide estimates of genetic diversity. **a** A comparison of nucleotide diversity among accessions from different countries. The “**” sign represents a significant difference (*P* < 0.01) between Senegalese landraces and the accessions from the given countries. **b** The distribution of inbreeding coefficients for global accessions and Senegalese landraces

**Fig. 4:**
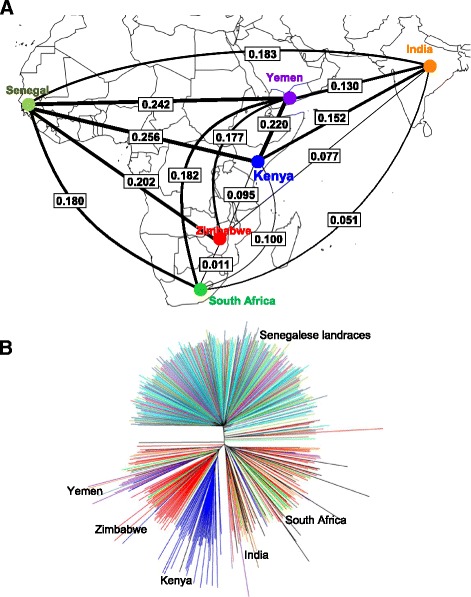
The genetic relatedness of pearl millet accessions. **a** F-statistic (*F*_*ST*_) between populations with different origins. The circles indicate the countries of origin, and the values represent the *F*_*ST*_ between accessions from the two countries. The thickness of the lines is proportional to the value of *F*_*ST*_. **b** Genetic relatedness among 500 accessions assessed with the neighbor joining method. Global accessions are colored by countries of origin, and Senegalese landraces are colored by regions of origin

The nucleotide diversity (π) of the whole panel was 0.224. The nucleotide diversity for global accessions (0.190) was slightly, but significantly, higher than that of Senegalese landraces (0.187) (*P*-value = 0.004). However, nucleotide diversity in other individual countries was lower than for the Senegalese landraces (Fig. 3a). The inbreeding coefficient of global accessions was significantly higher than that of Senegalese landraces (Fig. 3b). For differentiation coefficients (*F*_*ST*_) among countries, the values were higher between Senegalese landraces and any other countries (Fig. 4a). The lowest *F*_*ST*_ was observed between South Africa and Zimbabwe. The *F*_*ST*_ between global accessions and Senegalese landraces was 0.209.

To visualize the genetic relatedness among accessions, a neighbor-joining tree was generated. As shown in Fig. 4b, the accessions separated into two main groups, one group corresponding to the Senegalese landraces, another to the global accessions. Among the Senegalese landraces, there is little to no clustering corresponding to agro-ecological region. Among global accessions, distinct groups were observed for Yemen, Zimbabwe and Kenya, but accessions from India and South Africa were not distinctly clustered (Fig. 4b). A mixed group with lines from various countries was also observed.

### Population structure

**Fig. 5:**
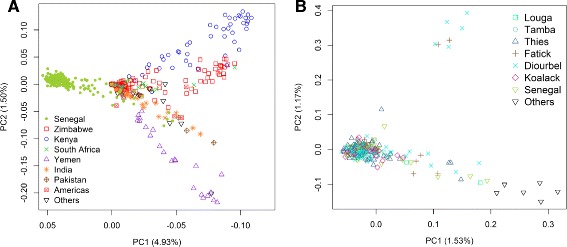
Population structure of global accessions and Senegalese landraces. **a** Principal component analysis for 500 pearl millet accessions (first two principal components), others represented all countries that were not listed. **b** Principal component analysis for 248 Senegalese landraces (first two principal components) when analyzed separately from the global accessions, others represented Niger, Mali, and Mauritania

We further characterized the population structure of pearl millet, globally and in Senegal, with principal component analysis (PCA). After filtering for missing data, linkage disequilibrium, and minor allele frequency, we obtained 8377 SNPs to conduct PCA on all accessions, and 7269 SNPs to conduct PCA on the Senegalese landraces. The total amount of genetic variation explained by first ten principal components (PCs) was 11.52 %. The first principal component (PC1), which explained 4.93 % of variation, separated the Senegalese landraces from the global accessions (Fig. 5a). The second PC (PC2), which explained 1.50 % of variation, reflected the geographical distribution of accessions, and separated southwest Asian germplasm (i.e. Yemen) from southeast African germplasm (i.e. South Africa, Zimbabwe and Kenya) (Fig. 5a). While there was extensive geographic structure among global accessions, there was little geographic structure in the Senegalese landraces (Fig. 5b).

**Fig. 6:**
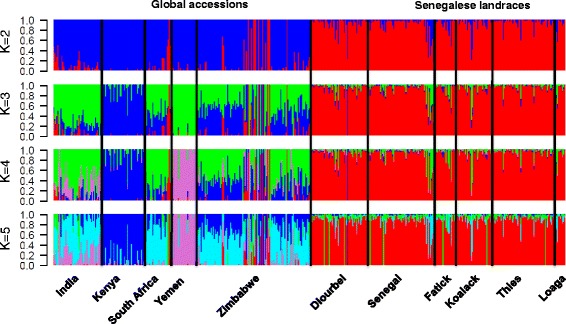
Ancestry estimates for pearl millet germplasm. Different colors represent the different subpopulations from ADMIXTURE, and text under the barplot indicates the origins of the corresponding germplasm. The K value left of the figures indicates the assumed number of subpopulations

Next, we investigated the population structure with model-based estimation of ancestry using ADMIXTURE. The lowest cross-validation error was observed when two genetic groups were assumed (*K* = 2) (Additional file 4: Figure S1), which divided the whole population into global accessions and Senegalese landraces (Fig. 6). A few individuals from the global accessions were assigned to the Senegalese landrace group, and some Senegalese landraces were classified into groups from the global accessions. No obvious geographic structure was found within Senegalese landraces when the number of groups increased. Global accessions separated into two subgroups (*K* = 3), one mainly from Yemen and Kenya, and another mainly from southern Africa and the rest of the world. At *K* = 5, the inbred lines were divided into three groups, corresponding to Yemen, Kenya, and southern Africa and India.

### Genomic organization of SNP variation

**Fig. 7:**
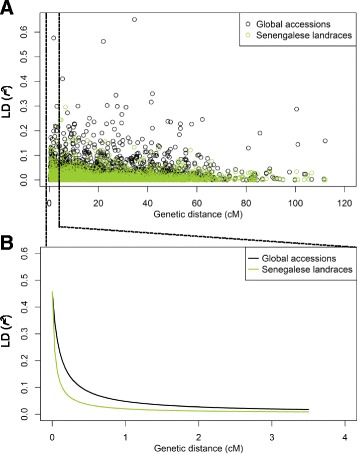
Linkage disequilibrium (LD) decay with respect to genetic distance. **a** Pairwise LD against genetic distance on all linkage groups for global accessions and Senegalese landraces. **b** LD against the genetic distance between 0 ~ 3.5 cM. The lines were plotted using non-linear model to fit the LD decay against the genetic distance

Although a complete physical map is not yet available for the pearl millet genome, insight into the genomic organization of SNP variation can be obtained using the genetic map. In order to assess genome-wide LD decay, we used genetic positions of 244 SNPs obtained from a previously-constructed linkage map [46] to calculate pairwise *r*^*2*^ for global accessions and Senegalese landraces, respectively (Fig. 7a). LD comparison of between Senegalese landraces and global accessions indicated substantially faster LD decay in Senegalese landraces versus global accessions (Fig. 7b).

**Fig. 8:**
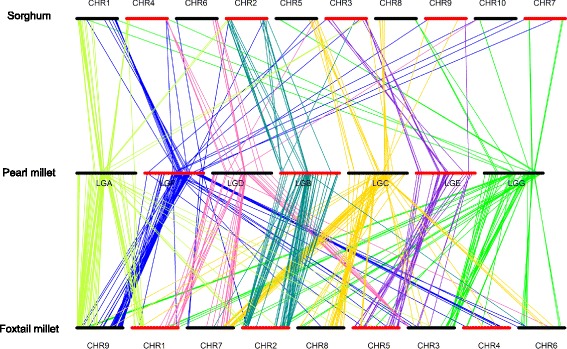
Synteny of pearl millet linkage groups with foxtail millet and sorghum genomes. “LG” represents the linkage groups of pearl millet, with a different line colors for each linkage group. The bottom bars represent the chromosomes of foxtail millet and the top bars represent the chromosomes of sorghum

To characterize the synteny of pearl millet with foxtail millet (*Setaria italica*) and sorghum (*Sorghum bicolor*), we performed a BLAST against the foxtail millet and sorghum genomes using the GBS tags that had been used to construct the genetic linkage maps [46]. More tags were mapped on the foxtail millet genome (267) than on the sorghum genome (92) (Fig. 8). The 267 pearl millet tags that mapped to foxtail millet corresponded to 370 genome positions, with 213 tags mapped to unique positions and 54 tags mapped to 2–6 positions. The 92 pearl millet tags that mapped to sorghum corresponded to 122 genome positions, with 73 tags mapped to unique positions and 19 tags mapped to 2–9 positions. The patterns of synteny reflect the closer relationship between pearl millet and foxtail millet as compared to sorghum, and point to large-scale rearrangements in the pearl millet genome after divergence with foxtail millet (Fig. 8).

## Discussion

### Effectiveness of GBS to characterize pearl millet genomic diversity

In the present study, we characterized 500 pearl millet accessions at 83,875 SNPs discovered and genotyped using a non-reference GBS approach [42]. For non-model species, the enzyme choice and GBS library construction strategy is important to balance the marker number and coverage [42]. The two enzyme (*PstI-MspI*) GBS approach is a powerful method for non-model species to identify high density markers [44]. The high outcrossing rates in pearl millet leads to high heterozygosity, which can be difficult to distinguish from sequencing error or mapping error. In order to further increase the sequencing coverage, we also conducted size selection during library construction to reduce genome representation and increase the depth of coverage at each locus [48]. Future studies with greater sequencing depth could increase the accuracy of SNP calls at heterozygous sites [49, 50].

Due to biased mutational processes in plant genomes (e.g. cytosine deamination), the ratio of transitions to transversions for bona fide polymorphisms is expected be much greater than one. Consistent with this expectation, the transition/transversion ratio for the pearl millet SNPs was 1.74 (Table 1). This ratio is somewhat lower than estimates from maize (2.5) [34] and *Arabidopsis* (2.4) [51]. The difference may be due to biased sampling of the genome based on the enzymes used in GBS or differences in efficacy of selection among the species [52]. Compared to previous studies, which identified little geographic structure [15, 29], extensive geographic structure was observed in global accessions in present study. This suggests that the greater number and density of markers obtained through GBS increased the power to identify moderate population structure. The germplasm used in present study spanned many regions of pearl millet cultivation, but sampling in the Sahelian region was limited to Senegal. While previous studies identified little population structure in germplasm from West Africa through to Sudan [15, 29], future research using genome-wide markers may reveal additional structure in this region.

### Effects of genetic bottlenecks on pearl millet diversity

During crop diffusion genetic bottlenecks can occur because of reduced effective population size, if only part of the genetic diversity introduced into the new regions and gene flow with other populations is restricted [53]. Here, we observed low nucleotide diversity in all other countries compared to Senegal, consistent with genetic bottlenecks during spread from West Africa and limited gene exchange among the secondary gene pools (Fig. 3) [54–56]. In addition, the greatest divergence observed was between Senegalese landrace and global accessions (Fig. 4). The relationship between geographic distance from West Africa and level of divergence suggests a major effect of isolation by distance (Fig. 4a). Although bottlenecks and isolation by distance appear to be the dominant processes at a genome scale, some novel variation may also have appeared during adaptation to new environments [57–59]. Further genome scans and association studies will be needed to identify loci responsible for adaptation during diffusion. Understanding the genetic basis of environmental adaptation in this crop may facilitate breeding for increased climate-resilience [60].

Differences in genetic diversity may also be in part due to the comparison of *in situ* diversity from farmer’s fields (Senegalese landraces) with *ex situ* collections from crop genebanks (global accessions). Indeed, the inbreeding coefficients of global accessions were substantially higher than the Senegalese landraces (Fig. 3b). Overall, though, the global accessions still contained slightly more genetic diversity than Senegalese landraces, reflecting the large number of countries across Africa, Asia, and the Americas that were represented in the global accessions.

### Implication for association studies and breeding in pearl millet

Population structure analysis of crops germplasm is used to guide germplasm utilization in breeding programs and design of QTL mapping experiment [53]. While geographic structure was observed among countries represented in the global accessions, the structure was observed within Senegalese germplasm did not present a geographic pattern (Figs. 3, 4, 5 and 6). The population structure in the Senegal collection (Figs. 5 and 6) is partly, but not entirely, due to the inclusion of improved or introduced materials (e.g., ISMI 9507, ISMI 9301, ICMV-IS 89305, ICMV-IS 99001, SOSAT-C88, and ICMV-IS 92222). Admixture with improved or wild pearl millets could also be involved [30, 31]. Understanding the extent of LD also provides information to guide genome wide association studies [55, 61, 62]. Because a pearl millet reference genome is not yet available, we used genetic distances obtained from a published genetic map [46] for a preliminary assessment of LD decay. We recovered 244 of the 314 PstI-MspI GBS markers from the linkage map in our experiment. This is likely due to minor differences in GBS library construction strategies: in the previous study only PCR-based complexity reduction step was used [46], while in our study additional electrophoretic size selection was used. The rapid LD decay in Senegalese landraces compared to global accessions (Fig. 7) likely reflects extensive gene flow within Senegal. The weak population structure and fast LD decay in the Senegalese landraces should make it a good population for genetic dissection of complex traits with genome wide association studies, given high density markers and a reference genome sequence.

### Evidence for rearrangement in the pearl millet genome

Analysis of synteny can provide insight into the structural evolution of plant genomes and help guide the development of genomic resources [63]. Although our synteny analysis is limited due to the small number of markers in the linkage map, we can still characterize conservation and rearrangement in pearl millet genome (Fig. 8). We observed extensive synteny between pearl millet and foxtail millet, and to a less extent between pearl millet and sorghum (Fig. 8). This is consistent with the known relationship among these species, and with previous comparative studies of their genetic and physical maps [64, 65]. Also consistent with previous findings, the comparisons of the pearl millet with foxtail millet and sorghum suggested that multiple large-scale rearrangements occurred in pearl millet lineage after divergence with foxtail millet [64, 66].

## Conclusions

We genotyped worldwide pearl millet germplasm with non-reference GBS to characterize genomic diversity, population structure, and synteny with other cereals. Our results demonstrated the value of GBS for rapid and cost-effective high-throughput genotyping of pearl millet diversity. In total, 83,875 SNPs were identified for 500 pearl millet accessions. Clear population structure was observed between global accessions and Senegalese landraces and geographic structure was observed among countries but not within Senegal. The comparative genomic analysis indicated extensive synteny between pearl millet and its panicoid cereal relatives, with multiple rearrangements in the pearl millet lineage. The data generated in present study will be a foundation for future genome wide association studies and genomics-assisted breeding in pearl millet.

## Methods

### Plant materials

Worldwide 511 pearl millet accessions, including a set of 249 pearl millet accessions contributed by the Senegalese Institute of Agricultural Research (Institut Sénégalais de Recherches Agricoles; ISRA) and 262 pearl millet accessions (Additional file 1: Table S1) provided by provided by USDA National Plant Germplasm System (NPGS) (http://www.ars-grin.gov), were used as plant materials. The 249 pearl millet accessions were collected by ISRA in 1992 to 1993 (Additional file 1: Table S1). Out of these accessions, 205 originate from four agro-ecological regions across Senegal (Fig. 1a) [47], while 38 do not have exact origin information in Senegal and six are introduced from Niger, Mali or Mauritania (Additional file 1: Table S1). In addition to landrace germplasm accessions of Senegalese origin, the materials tested also include 5 improved and 6 exotic pearl millets. The 262 global pearl millet accessions from the NPGS represented about 20 % of the total pearl millet collection held by the NPGS. These accessions mainly originated from African countries, such as Zimbabwe, South Africa, and Kenya, and South Asian countries, such as Yemen and India (see legend for Fig. 1b). Additionally, eight accessions were from the United States, one accession was from Brazil, three accessions were from Russia, and three accessions were without origin information. The country-level origins of the accessions were mapped with the “maps” package in R 3.1.2 [67].

### DNA extraction

The 262 global accessions were grown in a greenhouse of Kansas State University. Approximately 50 mg of leaf tissue from two seedlings for each accession were collected after 15 days of emergence. Each 96-well plate consisted of 95 lines and one blank as a control. The leaves were immediately freeze dried for 2 days utilizing a Labconco FreeZone (Labconco), and were grounded with a ball mill (Retsch). Genomic DNA was extracted with the Biosprint kit (QIAGEN). Two hundred forty nine Senegalese landraces were grown in experimental field in Senegal, and leaves were sampled after geminated. The DNA for 249 Senegalese accessions was extracted with MATAB protocol [68] at ISRA in Senegal, and sent to Kansas State University for further analysis. DNA for all the samples were quantified in plates with PicoGreen and DNA concentrations was normalized to 20 ng/ul with QIAgility robot (QIAGEN).

### GBS libraries construction and SNP calling

Two GBS libraries were generated, one multiplexing 380 lines with four blanks as control, and another multiplexing 285 lines with three blanks as control. GBS libraries were constructed following the two-enzyme (*PstI* and *MspI*) protocol developed by Poland et al. [39] with modification. For each plate a single random blank well was included for quality control. Genomic DNAs were co-digested with *PstI* (CTGCAG) and *MspI* (CCGC) restriction enzymes (New England Biolabs, Ipswitch, MA, USA), then barcoded adapters were ligated to each sample. Instead of NEB4 buffer CutSmart Buffer (New England Biolabs) was used in digestion and ligation. Samples were pooled by plate into a single library and purified with a QIAquick PCR purification kit (QIAGEN, Valencia CA, USA) before PCR amplification. Products in the size range from 250 to 400 bp (including adapter and primer sequence) were selected with the Pippin Prep system (Sage Science). All libraries were adjusted to 4 nM concentration. Four 96-plex libraries (SL1, SL2, SLGA1, and GA1) were pooled to form a 384-plex library (Lib_384) and three 96-plex libraries (SL3, SLGA2, and GA2) were pooled to form a 288-plex library (Lib_288) (Fig. 2a). The 384- and 288-plex libraries were sequenced with single-end Illumina HiSeq2500 (KU Medical Center, University of Kansas). These raw sequencing data have been submitted to the NCBI Short Read Achieve with accession numbers SRR2906941 and SRR2906969. SNP were identified using the TASSEL-UNEAK pipeline [42] in TASSEL 4 package [69], −*c* option for minimum count of a tag was set as 5, −*e* option for error tolerance rate was set as 0.03. The Hapmap format data was converted to VCF format with TASSEL 4 [69] for following analysis.

### Population genetic analysis

The minor allele frequency was analyzed with TASSEL 4 [69]. The neutral allele expectation distribution was conducted with the methods that developed by Fu, Griffiths and Tavaré [70, 71]. The nucleotide diversity (π) and inbreeding coefficients for each population, and the *F*_*ST*_ between populations were calculated using vcftools v0.1.13 [72]. The genetic diversity was compared between global accessions and Senegalese landraces with *t*-test in R 3.1.2 [67].

Two approaches were used to infer the population structure. First, Principal Component Analysis (PCA) was carried out using SNPRelate_1.0.1 R package [73]. The *snpgdsLDpruning* function was used to filter the SNP data with following options: *method = “corr”, ld.threshold = 0.5, maf = 0.05, missing = 0.8*. The *snpgdsPCA* function was used to conduct PCA with default setting. The SNP data used for PCA was extracted with custom R script and was used for the second population structure analysis method, a model based estimate of ancestry, which was carried out using the ADMIXTURE 1.23 [74], with the number of genetic groups range from *K* = 1 to *K* = 10. The optimal number of subpopulations was determined with lowest Cross-validation error. The VCF format data was converted to data format required by ADMIXTURE 1.23 using PLINK 1.07 [75].

The genetic distance matrix between individuals was generated and neighbor joining tree was constructed using TASSEL 4 [69], and was visualized with APE 3.0-11 package [76]. Linkage disequilibrium (LD) decay of global accessions and Senegalese landraces was compared based on previous genetics map [46]. Due to lack of reference genome, we mapped the SNP tags of our data on the tags that have been mapped on linkage maps [46] that was constructed with PstI-MspI GBS to obtain the genetic position. The LD of each pair of SNPs was calculated using TASSEL 4 [50].

### Comparative genomic with foxtail millet and sorghum

The reference genomes for foxtail millet and sorghum (v1.4) were downloaded from BGI-Shenzhen (http://foxtailmillet.genomics.org.cn/) [66] and Phytozome (http://phytozome.jgi.doe.gov) [77, 78], respectively. SNP tags from linkage maps [46] were mapped to the reference genomes using Basic Local Alignment Search Tool (BLAST) [79] with a E-value cutoff of 1e-5. Synteny between pearl millet and foxtail millet, sorghum were visualized with custom R scripts [67].

### Ethics

No research involving human subjects, human data, or regulated vertebrates or invertebrates was included in this study.

## Additional files

Additional file 1: Table S1. The germplasm used in this study. (XLSX 46 kb) Additional file 2: Table S2. Genotyping by sequencing data and SNP statistics. (DOCX 13 kb) Additional file 3: Figure S2. Minor allele frequency distribution for Senegalese landraces (A) and global accessions (B). The red lines represent the expected neutral allele frequency distribution. (PPTX 187 kb) Additional file 4: Figure S1. Cross-validation error for model-based ancestry estimates. The cross-validation error is lowest when K = 2. (PPTX 63 kb)

## Abbreviations

AFLP: Amplified fragment length polymorphism
BLAST: Basic Local Alignment Search Tool
GBS: Genotyping-by-sequencing
LD: Linkage disequilibrium
PCA: Principal components analysis
QTL: Quantitative trait loci
RFLP: Restriction fragment length polymorphism
SNP: Single-nucleotide polymorphism
UNEAK: Universal network enable analysis kit
VCF: Variant call format

## References

[1] Lobell DB, Burke MB, Tebaldi C, Mastrandrea MD, Falcon WP, Naylor RL (2008). Prioritizing climate change adaptation needs for food security in 2030. Science.

[2] Wheeler T, von Braun J (2013). Climate change impacts on global food security. Science.

[3] National Research Council (1996). Lost crops of Africa: Volume I: Grains.

[4] Saleh ASM, Zhang Q, Chen J, Shen Q (2013). Millet grains: Nutritional quality, processing, and potential health benefits. Compr Rev Food Sci Food Saf.

[5] Campbell BM, Thornton P, Zougmoré R, van Asten P, Lipper L (2014). Sustainable intensification: What is its role in climate smart agriculture?. Curr Opin Environ Sustain.

[6] Varshney RK, Ribaut J-M, Buckler ES, Tuberosa R, Rafalski JA, Langridge P (2012). Can genomics boost productivity of orphan crops?. Nat Biotechnol.

[7] Brunken JN (1977). A systematic study of Pennisetum sect. Pennisetum (Gramineae). Am J Bot.

[8] Harlan JR (1975). Crops & man.

[9] Manning K, Pelling R, Higham T, Schwenniger J-L, Fuller DQ (2011). 4500-Year old domesticated pearl millet (Pennisetum glaucum) from the Tilemsi Valley, Mali: New insights into an alternative cereal domestication pathway. J Archaeol Sci.

[10] Ardlie KG, Kruglyak L, Seielstad M (2002). Patterns of linkage disequilibrium in the human genome. Nat Rev Genet.

[11] Oumar I, Mariac C, Pham J-L, Vigouroux Y (2008). Phylogeny and origin of pearl millet (Pennisetum glaucum [L.] R. Br) as revealed by microsatellite loci. Theor Appl Genet.

[12] Tostain S, Marchais L (1989). Enzyme diversity in pearl millet (Pennisetum glaucum). Theor Appl Genet.

[13] Evenson RE, Gollin D (2003). Crop Variety Improvement and Its Effect on Productivity: The Impact of International Agricultural Research.

[14] Busso CS, Devos KM, Ross G, Mortimore M, Adams WM, Ambrose MJ (2000). Genetic diversity within and among landraces of pearl millet (Pennisetum glaucum) under farmer management in West Africa. Genet Resour Crop Evol.

[15] Stich B, Haussmann BI, Pasam R, Bhosale S, Hash CT, Melchinger AE (2010). Patterns of molecular and phenotypic diversity in pearl millet [Pennisetum glaucum (L.) R. Br.] from West and Central Africa and their relation to geographical and environmental parameters. BMC Plant Biol.

[16] Jin L, Lu Y, Xiao P, Sun M, Corke H, Bao J (2010). Genetic diversity and population structure of a diverse set of rice germplasm for association mapping. Theor Appl Genet.

[17] Kilian B, Graner A (2012). NGS technologies for analyzing germplasm diversity in genebanks. Brief Funct Genomics.

[18] Jones ES, Breese WA, Liu CJ, Singh SD, Shaw DS, Witcombe JR (2002). Mapping quantitative trait loci for resistance to downy mildew in pearl millet. Crop Sci.

[19] Kannan B, Senapathy S, Bhasker Raj AG, Chandra S, Muthiah A, Dhanapal AP (2014). Association Analysis of SSR markers with phenology, grain, and stover-yield related Traits in pearl millet (*Pennisetum glaucum* (L.) R. Br.). Sci World J.

[20] Mariac C, Jehin L, Saïdou A-A, Thuillet A-C, Couderc M, Sire P (2011). Genetic basis of pearl millet adaptation along an environmental gradient investigated by a combination of genome scan and association mapping. Mol Ecol.

[21] Parvathaneni RK, Jakkula V, Padi FK, Faure S, Nagarajappa N, Pontaroli AC (2013). Fine-mapping and identification of a candidate gene underlying the d2 dwarfing phenotype in pearl millet, Cenchrus americanus (L.) Morrone. G3.

[22] Qi X, Pittaway TS, Lindup S, Liu H, Waterman E, Padi FK (2004). An integrated genetic map and a new set of simple sequence repeat markers for pearl millet, Pennisetum glaucum. Theor Appl Genet.

[23] Sehgal D, Rajaram V, Armstead IP, Vadez V, Yadav YP, Hash CT (2012). Integration of gene-based markers in a pearl millet genetic map for identification of candidate genes underlying drought tolerance quantitative trait loci. BMC Plant Biol.

[24] Supriya A, Senthilvel S, Nepolean T, Eshwar K, Rajaram V, Shaw R (2011). Development of a molecular linkage map of pearl millet integrating DArT and SSR markers. Theor Appl Genet.

[25] Yadav RS, Sehgal D, Vadez V (2011). Using genetic mapping and genomics approaches in understanding and improving drought tolerance in pearl millet. J Exp Bot.

[26] Wu J, Li L-T, Li M, Khan MA, Li X-G, Chen H (2014). High-density genetic linkage map construction and identification of fruit-related QTLs in pear using SNP and SSR markers. J Exp Bot.

[27] Bhattacharjee R, Bramel J, Hash T, Kolesnikova-Allen A, Khairwal S (2002). Assessment of genetic diversity within and between pearl millet landraces. Theor Appl Genet.

[28] Brocke KV, Christinck A, Weltzien E, Presterl T, Geiger HH (2003). Farmers seed systems and management practices determine pearl millet genetic diversity patterns in semiarid regions of India. Crop Sci.

[29] Bashir EMA, Ali AM, Ali AM, Mohamed ETI, Melchinger AE, Parzies HK (2014). Genetic diversity of Sudanese pearl millet (Pennisetum glaucum (L.) R. Br.) landraces as revealed by SSR markers, and relationship between genetic and agro-morphological diversity. Genet Resour Crop Evol.

[30] Budak H, Pedraza F, Cregan PB, Baenziger PS, Dweikat I (2003). Development and Utilization of SSRs to Estimate the Degree of Genetic Relationships in a Collection of Pearl Millet Germplasm. Crop Sci.

[31] Mariac C, Luong V, Kapran I, Mamadou A, Sagnard F, Deu M (2006). Diversity of wild and cultivated pearl millet accessions (Pennisetum glaucum [L.] R. Br.) in Niger assessed by microsatellite markers. Theor Appl Genet.

[32] Nepolean T, Gupta SK, Dwivedi SL, Bhattacharjee R, Rai KN, Hash CT (2012). Genetic Diversity in Maintainer and Restorer Lines of Pearl Millet. Crop Sci.

[33] Huang X, Kurata N, Wei X, Wang Z-X, Wang A, Zhao Q (2012). A map of rice genome variation reveals the origin of cultivated rice. Nature.

[34] Jiao Y, Zhao H, Ren L, Song W, Zeng B, Guo J (2012). Genome-wide genetic changes during modern breeding of maize. Nat Genet.

[35] Wu P, Zhou C, Cheng S, Wu Z, Lu W, Han J (2015). Integrated genome sequence and linkage map of physic nut (Jatropha curcas L.), a biodiesel plant. Plant J.

[36] Zhou Z, Jiang Y, Wang Z, Gou Z, Lyu J, Li W (2015). Resequencing 302 wild and cultivated accessions identifies genes related to domestication and improvement in soybean. Nat Biotechnol.

[37] Miller MR, Dunham JP, Amores A, Cresko WA, Johnson EA (2007). Rapid and cost-effective polymorphism identification and genotyping using restriction site associated DNA (RAD) markers. Genome Res.

[38] Elshire RJ, Glaubitz JC, Sun Q, Poland JA, Kawamoto K, Buckler ES (2011). A Robust, simple genotyping-by-sequencing (GBS) approach for high diversity species. PLoS One.

[39] Poland J, Endelman J, Dawson J, Rutkoski J, Wu S, Manes Y, Dreisigacker S, Crossa J, Sánchez-Villeda H, Sorrells M, Jannink J-L: Genomic Selection in Wheat Breeding using Genotyping-by-Sequencing. *Plant Genome* 2012, 5:103:10.3835/plantgenome2012.06.0006.

[40] Bastien M, Sonah H, Belzile F. Genome Wide Association Mapping of Resistance in Soybean with a Genotyping-by-Sequencing Approach. *Plant Genome* 2014, 7:0:10.3835/plantgenome2013.10.0030.

[41] Jarquín D, Kocak K, Posadas L, Hyma K, Jedlicka J, Graef G (2014). Genotyping by sequencing for genomic prediction in a soybean breeding population. BMC Genomics.

[42] Lu F, Lipka AE, Glaubitz J, Elshire R, Cherney JH, Casler MD (2013). Switchgrass genomic diversity, ploidy, and evolution: Novel insights from a network-based SNP discovery protocol. PLoS Genet.

[43] Ramu P, Deshpande SP, Hash CT, Shah T, Upadhyaya HD, Riera-Lizarazu O (2013). Population genomic and genome-wide association studies of agroclimatic traits in sorghum. Proc Natl Acad Sci.

[44] Poland JA, Brown PJ, Sorrells ME, Jannink J-L (2012). Development of high-density genetic maps for barley and wheat using a novel two-enzyme genotyping-by-sequencing approach. PLoS One.

[45] Romay MC, Millard MJ, Glaubitz JC, Peiffer JA, Swarts KL, Casstevens TM (2013). Comprehensive genotyping of the USA national maize inbred seed bank. Genome Biol.

[46] Moumouni KH, Kountche BA, Jean M, Hash CT, Vigouroux Y, Haussmann BIG (2015). Construction of a genetic map for pearl millet, Pennisetum glaucum (L.) R. Br., using a genotyping-by-sequencing (GBS) approach. Mol Breed.

[47] Tappan GG, Sall M, Wood EC, Cushing M (2004). Ecoregions and land cover trends in Senegal. J Arid Environ.

[48] Beissinger TM, Hirsch CN, Sekhon RS, Foerster JM, Johnson JM, Muttoni G (2013). Marker density and read depth for genotyping populations using genotyping-by-sequencing. Genetics.

[49] Bryc K, Patterson N, Reich D (2013). A novel approach to estimating heterozygosity from low-coverage genome sequence. Genetics.

[50] Glaubitz JC, Casstevens TM, Lu F, Harriman J, Elshire RJ, Sun Q (2014). TASSEL-GBS: A high capacity genotyping by sequencing analysis pipeline. PLoS One.

[51] Ossowski S, Schneeberger K, Lucas-Lledó JI, Warthmann N, Clark RM, Shaw RG (2010). The rate and molecular spectrum of spontaneous mutations in Arabidopsis thaliana. Science.

[52] Keller I, Bensasson D, Nichols RA (2007). Transition-transversion bias is not universal: A counter example from grasshopper pseudogenes. PLoS Genet.

[53] Morrell PL, Buckler ES, Ross-Ibarra J (2012). Crop genomics: Advances and applications. Nat Rev Genet.

[54] Briggs WH, Goldman IL (2006). Genetic variation and selection response in model breeding populations of Brassica rapa following a diversity Bottleneck. Genetics.

[55] Doebley JF, Gaut BS, Smith BD (2006). The molecular genetics of crop domestication. Cell.

[56] Hyten DL, Song Q, Zhu Y, Choi I-Y, Nelson RL, Costa JM (2006). Impacts of genetic bottlenecks on soybean genome diversity. Proc Natl Acad Sci.

[57] Buckley J, Bridle JR, Pomiankowski A (2010). Novel variation associated with species range expansion. BMC Evol Biol.

[58] Rainey PB, Travisano M (1998). Adaptive radiation in a heterogeneous environment. Nature.

[59] Wootton JC, Feng X, Ferdig MT, Cooper RA, Mu J, Baruch DI (2002). Genetic diversity and chloroquine selective sweeps in Plasmodium falciparum. Nature.

[60] Kole C, Muthamilarasan M, Henry R, Edwards D, Sharma R, Abberton M (2015). Application of genomics-assisted breeding for generation of climate resilient crops: Progress and prospects. Plant Genet Genomics.

[61] Allouis S, Qi X, Lindup S, Gale MD, Devos KM (2001). Construction of a BAC library of pearl millet, Pennisetum glaucum. Theor Appl Genet.

[62] Slatkin M (2008). Linkage disequilibrium--understanding the evolutionary past and mapping the medical future. Nat Rev Genet.

[63] Mayer KFX, Martis M, Hedley PE, Šimková H, Liu H, Morris JA (2011). Unlocking the Barley genome by chromosomal and comparative genomics. Plant Cell.

[64] Devos KM, Pittaway TS, Reynolds A, Gale MD (2000). Comparative mapping reveals a complex relationship between the pearl millet genome and those of foxtail millet and rice. Theor Appl Genet.

[65] Rajaram V, Nepolean T, Senthilvel S, Varshney RK, Vadez V, Srivastava RK (2013). Pearl millet [Pennisetum glaucum (L.) R. Br.] consensus linkage map constructed using four RIL mapping populations and newly developed EST-SSRs. BMC Genomics.

[66] Zhang G, Liu X, Quan Z, Cheng S, Xu X, Pan S (2012). Genome sequence of foxtail millet (Setaria italica) provides insights into grass evolution and biofuel potential. Nat Biotechnol.

[67] R Core Team (2014). R: A Language and environment for statistical computing.

[68] Risterucci AM, Grivet L, N’Goran JAK, Pieretti I, Flament MH, Lanaud C (2000). A high-density linkage map of Theobroma cacao L. Theor Appl Genet.

[69] Bradbury PJ, Zhang Z, Kroon DE, Casstevens TM, Ramdoss Y, Buckler ES (2007). TASSEL: Software for association mapping of complex traits in diverse samples. Bioinformatics.

[70] Fu YX (1995). Statistical properties of segregating sites. Theor Popul Biol.

[71] Griffiths RC, Tavaré S (1998). The age of a mutation in a general coalescent tree. Commun Stat Stoch Models.

[72] Danecek P, Auton A, Abecasis G, Albers CA, Banks E, DePristo MA (2011). The variant call format and VCFtools. Bioinformatics.

[73] Zheng X, Levine D, Shen J, Gogarten SM, Laurie C, Weir BS (2012). A high-performance computing toolset for relatedness and principal component analysis of SNP data. Bioinformatics.

[74] Alexander DH, Novembre J, Lange K (2009). Fast model-based estimation of ancestry in unrelated individuals. Genome Res.

[75] Purcell S, Neale B, Todd-Brown K, Thomas L, Ferreira MAR, Bender D (2007). PLINK: A tool set for whole-genome association and population-based linkage analyses. Am J Hum Genet.

[76] Paradis E, Claude J, Strimmer K (2004). APE: Analyses of phylogenetics and evolution in R language. Bioinformatics.

[77] Goodstein DM, Shu S, Howson R, Neupane R, Hayes RD, Fazo J (2012). Phytozome: A comparative platform for green plant genomics. Nucleic Acids Res.

[78] Paterson AH, Bowers JE, Bruggmann R, Dubchak I, Grimwood J, Gundlach H (2009). The Sorghum bicolor genome and the diversification of grasses. Nature.

[79] Altschul SF, Madden TL, Schäffer AA, Zhang J, Zhang Z, Miller W (1997). Gapped BLAST and PSI-BLAST: A new generation of protein database search programs. Nucleic Acids Res.

