# Supplementary material for: Population genomics of pearl millet (Pennisetum glaucum (L.) R. Br.): Comparative analysis of global accessions and Senegalese landraces
Source: BMC Genomics. 2015 Dec 9;16:1048. doi: 10.1186/s12864-015-2255-0 (PMC4674952; doi:10.1186/s12864-015-2255-0)
Supplement: Additional file 2: Table S2. — Genotyping by sequencing data and SNP statistics. (DOCX 13 kb) [file 12864_2015_2255_MOESM2_ESM.docx]

Table S2 Genotyping by sequencing data and SNP statistics

| Statistics types | Results/Criteria |
| --- | --- |
| Raw data | ~110 GB |
| Read number | 208 M (Lib_384) and 217 M Lib_288 |
| Rate of read with barcode | Lib_384: 88.3% and Lib_288: 39.0% |
| Average count number of tag per site | 1264.75 |
| Average missing number and rate for SNP | 323.80 and 0.65% |
| Average missing number and rate for individual | 54316.73 and 0.65% |
| SNP number | 83, 875 |
| Eleven genotypes removed | < 6 SNPs |
| Number of allele per site | Two |
