# Supplementary figures and images for: Population genomics of pearl millet (Pennisetum glaucum (L.) R. Br.): Comparative analysis of global accessions and Senegalese landraces
Source: BMC Genomics. 2015 Dec 9;16:1048. doi: 10.1186/s12864-015-2255-0 (PMC4674952; doi:10.1186/s12864-015-2255-0)

## Slide 1
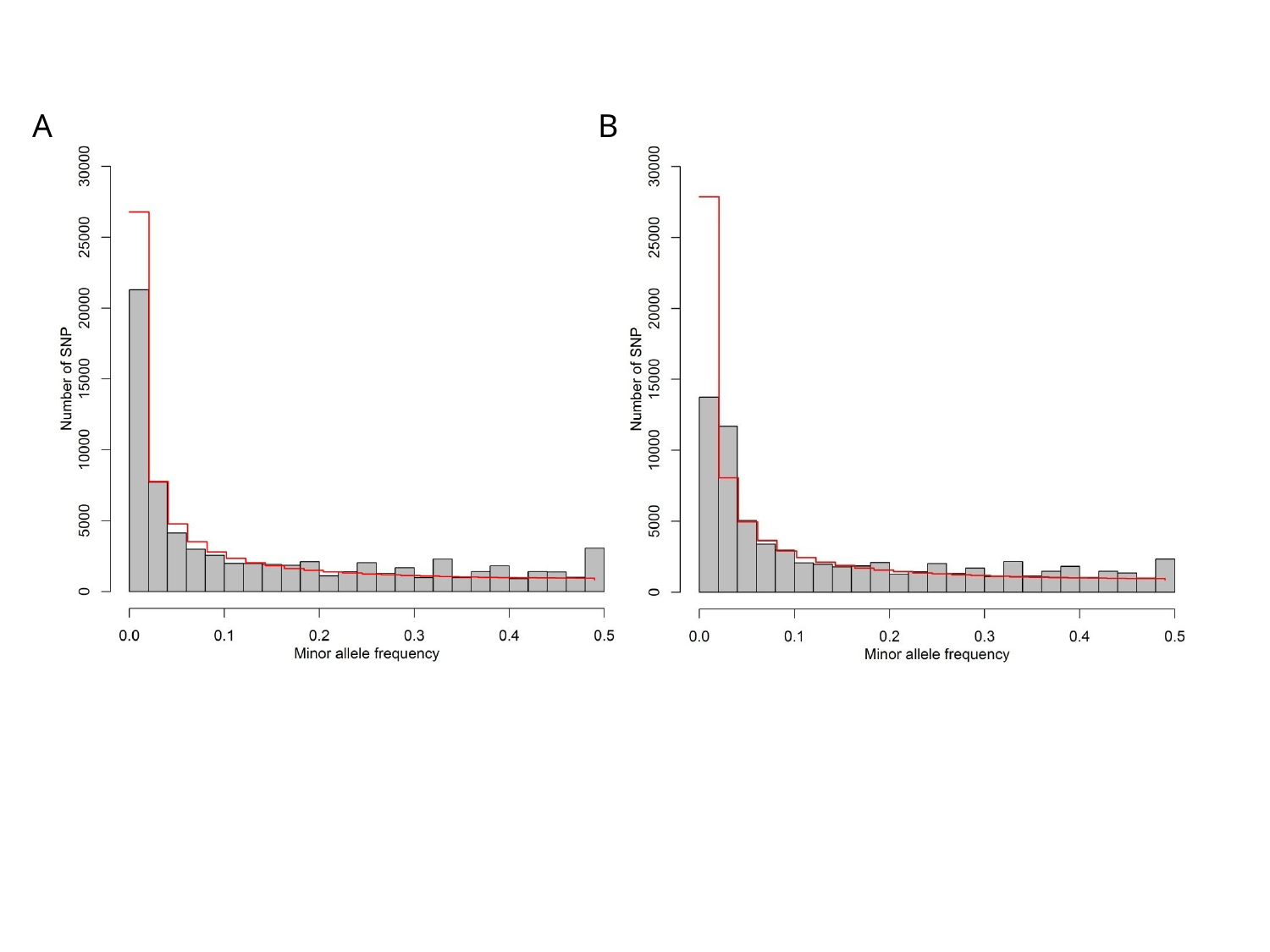

A
B

Supplement: Additional file 3: Figure S2. — Minor allele frequency distribution for Senegalese landraces (A) and global accessions (B). The red lines represent the expected neutral allele frequency distribution. (PPTX 187 kb) [file 12864_2015_2255_MOESM3_ESM.pptx]

## Slide 1
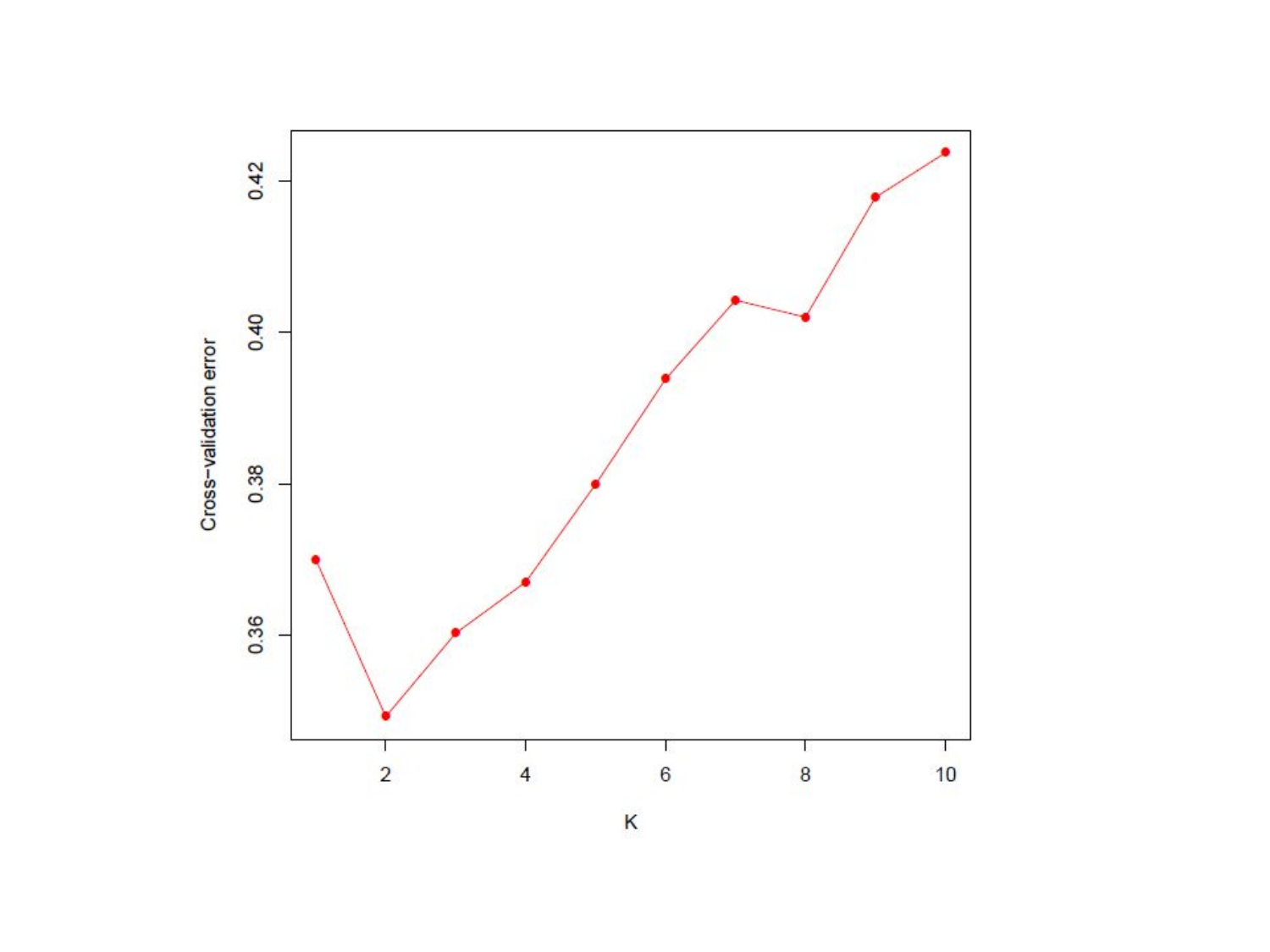

Supplement: Additional file 4: Figure S1. — Cross-validation error for model-based ancestry estimates. The cross-validation error is lowest when K = 2. (PPTX 63 kb) [file 12864_2015_2255_MOESM4_ESM.pptx]
